# Supplementary material for: Association between the Composite Dietary Antioxidant Index and Atherosclerotic Cardiovascular Disease in Postmenopausal Women: A Cross-Sectional Study of NHANES Data, 2013–2018
Source: Antioxidants (Basel). 2023 Sep 8;12(9):1740. doi: 10.3390/antiox12091740 (PMC10525155; doi:10.3390/antiox12091740)
Supplement: Supplementary file 1 [file antioxidants-12-01740-s001.zip › antioxidants-2541891-supplementary.pdf]

SUPPLEMENTARY TABLE S1 Description of Covariates

**Covariates**	**Description**
Age	For individuals who were 80 years and older, their age was topcoded at 80 years. Age was categorized into five groups: 40-49, 50-59, 60-69, 70-79, and ⩾80 years.
Race	Race was reported as Mexican American, other Hispanic, non-Hispanic white, non-Hispanic black, or other race.
Education level	Education level was categorized as less than high school, high school or equivalent, or college or above.
Marital status	Marital status was reported as married, widowed, divorced, separated, never married, or living with a partner.
Ratio of family income to poverty	Ratio of family income to poverty was divided into three groups: ⩽1.00, 1.01-3.00, and >3.00.
The body mass index (BMI)	The body mass index (BMI) was calculated by dividing weight in kilograms by height in meters squared. It was categorized into four groups: underweight (<18.5 kg/m^2), normal weight (18.5-24.9 kg/m^2), overweight (25.0-29.9 kg/m^2), and obesity (⩾30 kg/m^2).
Alcohol use	Alcohol use was defined as consuming at least 12 drinks of any type of alcoholic beverage in any one year. Alcoholic beverages included liquor, beer, wine, wine coolers, and any other type of alcoholic beverage.

Smoking-cigarette use	Smoking behaviors were based on whether participants had smoked at least 100 cigarettes in their lifetime.
Moderate to vigorous recreational activities	The Physical Activity questionnaire recorded whether participants engaged in moderate or vigorous recreational activities. Responses were categorized as “yes” or “no”.
Sleep disorders	Participants were asked whether they had ever told a doctor that they had trouble sleeping, and those who answered “yes” were classified as having sleep disorders.
Hypertension	Hypertension was defined based on self-reported information, either from a doctor’s diagnosis or advice to take antihypertensive medication.
Diabetes	Diabetes was defined as self-reported diabetes (participants who answered “yes” to the question “Has a doctor told you that you have diabetes?”) or current use of hypoglycemic agents or insulin, or a hemoglobin A1c (HbA1c) level $\geqslant$ 6.5%.
A family history of heart	A family history of heart attack was defined as a self-reported “yes” response to the question “Have any of your close biological relatives, including father, mother, sisters or brothers, been told by a health professional that they had a heart attack or angina before the age of 50?”.
Neutrophil-to-	Lymphocyte and neutrophil counts were assessed using

lymphocyte ratio (NLR)	automated hematology analysis devices and expressed as $\times 1{,}000$ cells/mm^3. The neutrophil-to-lymphocyte ratio (NLR) was calculated as the ratio of the neutrophil count to the lymphocyte count.
Total daily caloric intake	Total daily caloric intake was estimated by analyzing the types and amounts of food and beverages (including all types of water) consumed during the 24-hour period preceding the interview (midnight to midnight).
Total daily polyunsaturated fatty acids	The dietary intake data are used to estimate the types and amounts of foods and beverages (including all types of water) consumed during the 24-hour period prior to the interview (midnight to midnight), and to estimate intakes of energy, nutrients, and other food components from those foods and beverages.

SUPPLEMENTARY TABLE S2 The Associations between CDAI Levels and the Risks for ASCVD and Hard Criteria in Five Groups of Postmenopausal Women by Logistic Regression [a]

CDAI	Group A		Group B		Group C		Group D		Group E	
	ASCVD	Hard criteria	ASCVD	Hard criteria	ASCVD	Hard criteria	ASCVD	Hard criteria	ASCVD	Hard criteria
**As continuous (per SD)**	0.68 (0.64, 0.84)**	0.75 (0.64, 0.92)*	0.59 (0.36, 0.99)*	0.56 (0.34, 0.94)*	0.67 (0.47, 0.95)**	0.61 (0.44, 0.85)**	0.64 (0.47, 0.87)**	0.65 (0.46, 0.87)**	0.66 (0.48, 0.89)**	0.59 (0.41, 0.85)**
**By cut-off**										
Low	Ref.	Ref.	Ref.	Ref.	Ref.	Ref.	Ref.	Ref.	Ref.	Ref.
High	0.70 (0.47, 0.95)*	0.60 (0.40, 0.94)*	0.58 (0.30, 0.86)**	0.71 (0.47, 1.05)	0.53 (0.32, 0.89)*	0.60 (0.38, 0.94)**	0.59 (0.39, 0.89)*	0.55 (0.39, 0.78)**	0.64 (0.44, 0.94)*	0.60 (0.39, 0.91)*
**Interquartile**										
Quartile 1	Ref.	Ref.	Ref.	Ref.	Ref.	Ref.	Ref.	Ref.	Ref.	Ref.

Quartile 2	0.63 (0.44, 0.90)*	0.73 (0.40, 1.35)	0.64 (0.34, 1.18)	0.81 (0.39, 1.49)	0.71 (0.40, 1.27)	0.71 (0.33, 1.26)	0.68 (0.47, 0.99)*	0.75 (0.48, 1.19)	0.68 (0.48, 0.97)*	0.76 (0.48, 1.21)
Quartile 3	0.64 (0.43, 0.97)*	0.63 (0.38, 1.01)	0.55 (0.21, 1.01)	0.70 (0.25, 1.36)	0.54 (0.25, 1.08)	0.51 (0.30, 0.85)*	0.58 (0.36, 0.92)*	0.63 (0.39, 0.97)*	0.61 (0.38, 0.94)*	0.65 (0.38, 1.03)
Quartile 4	0.52 (0.27, 0.98)*	0.28 (0.10, 0.77)**	0.36 (0.10, 0.71)**	0.13 (0.04, 0.45)**	0.49 (0.27, 0.89)**	0.36 (0.18, 0.73)**	0.48 (0.24, 0.95)*	0.33 (0.16, 0.64)**	0.46 (0.22, 0.95)*	0.29 (0.11, 0.77)*
p-trend	0.021	0.013	<0.001	0.031	0.029	0.003	0.036	0.001	0.042	0.015

Abbreviations: ACE, angiotensin-converting enzyme; ASCVD, atherosclerotic cardiovascular disease; BMI, body mass index; CDAI, composite dietary antioxidant index; CI, confidence interval; HDL-C, high-density lipoprotein cholesterol; NLR, neutrophil-to-lymphocyte ratio; OR, odds ratio; SGLT2, sodium-glucose cotransporter-2.

[a] The ORs (95%CI) were used to compare the associations between CDAI levels and the risks for ASCVD and hard criteria among five groups of postmenopausal women: those who never used female hormones (Group A), those who currently not taking antihypertensive (Group B), lipid-lowering (Group C), or antidiabetic medication (Group D), and individuals who had used statins, SGLT2 inhibitors, or ACE inhibitors within one month prior to their interview date (Group E). Female hormone use was defined as individuals who had ever used any forms of female hormones, including pills, cream, patch, and injectables, but not for birth control or infertility purposes. All models adjusted for age, race, education level, marital status, the ratio of family income to poverty, BMI, waist circumference, alcohol use, smoking-cigarette use, moderate to vigorous recreational activities, sleep disorders, hypertension, diabetes, a family history of heart attack, NLR, HDL-C, total cholesterol, total daily caloric intake, and total daily polyunsaturated fatty acids.

*$p \leq 0.05$; **$p \leq 0.01$; ***$p \leq 0.001$.
